# Muscles in Winter: The Epigenetics of Metabolic Arrest

**DOI:** 10.3390/epigenomes5040028

**Published:** 2021-12-16

**Authors:** W. Aline Ingelson-Filpula, Kenneth B. Storey

**Affiliations:** Department of Biology, Carleton University, 1125 Colonel By Drive, Ottawa, ON K1S 5B6, Canada; alineingelsonfilpula@cmail.carleton.ca

**Keywords:** muscle, metabolic rate depression, hypoxia, anoxia, freeze tolerance, torpor, hibernation, cold-hardiness

## Abstract

The winter months are challenging for many animal species, which often enter a state of dormancy or hypometabolism to “wait out” the cold weather, food scarcity, reduced daylight, and restricted mobility that can characterize the season. To survive, many species use metabolic rate depression (MRD) to suppress nonessential metabolic processes, conserving energy and limiting tissue atrophy particularly of skeletal and cardiac muscles. Mammalian hibernation is the best recognized example of winter MRD, but some turtle species spend the winter unable to breathe air and use MRD to survive with little or no oxygen (hypoxia/anoxia), and various frogs endure the freezing of about two-thirds of their total body water as extracellular ice. These winter survival strategies are highly effective, but create physiological and metabolic challenges that require specific biochemical adaptive strategies. Gene-related processes as well as epigenetic processes can lower the risk of atrophy during prolonged inactivity and limited nutrient stores, and DNA modifications, mRNA storage, and microRNA action are enacted to maintain and preserve muscle. This review article focuses on epigenetic controls on muscle metabolism that regulate MRD to avoid muscle atrophy and support winter survival in model species of hibernating mammals, anoxia-tolerant turtles and freeze-tolerant frogs. Such research may lead to human applications including muscle-wasting disorders such as sarcopenia, or other conditions of limited mobility.

## 1. Introduction

**Figure 1:**
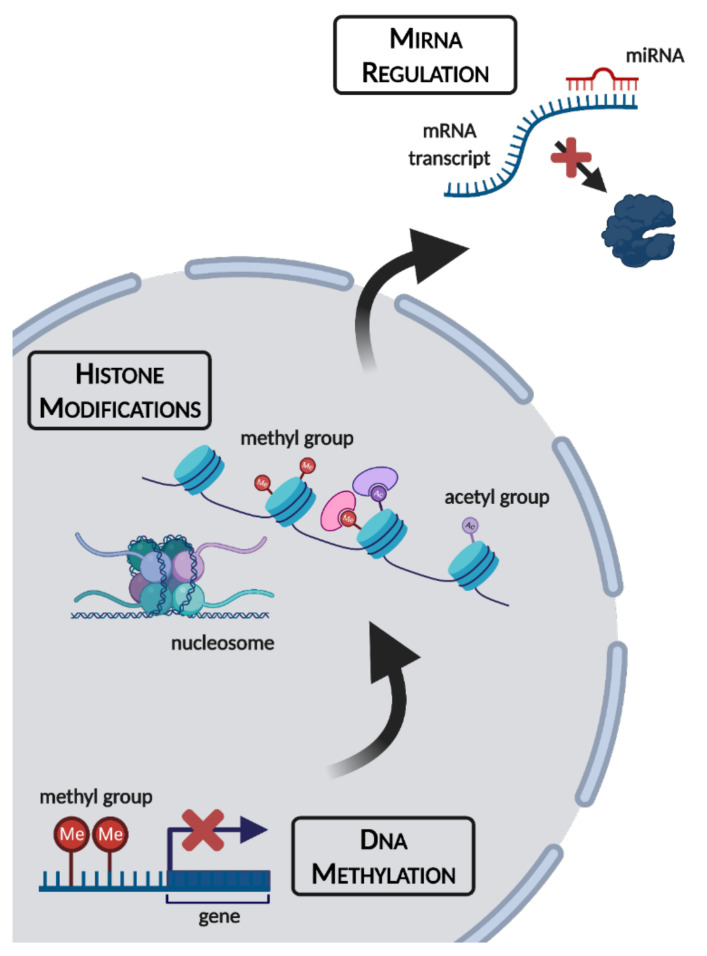
An overview of epigenetic mechanisms and their cellular locations. Figure made using BioRender.com (accessed on 7 October 2021).

As humans, we have the unique ability to manipulate our environment to suit our comfort levels and physiological needs. During the winter, technological advances including robust food supply chains and central heating ensure that we need not be subjected to starvation or subzero temperatures for extended periods of time. For many animal species, this is not the case. Seasonal conditions can vary drastically, with winter bringing a myriad of extreme environmental stresses including shorter photoperiods, subzero temperatures, and food scarcity. Some species migrate to avoid the worst of these conditions, but non-migratory species have evolved defensive strategies including hibernation, freezing survival, and hypoxia/anoxia tolerance to stave off the worst of winter’s influences [1,2,3,4]. All of these approaches have been documented in species ranging from invertebrates such as soil microfauna, insects, and intertidal marine molluscs to vertebrates including endothermic mammals and ectothermic species such as turtles and frogs [4,5,6,7,8,9]. Extreme survival responses are possible thanks in part to the phenomenon of metabolic rate depression (MRD), characterized by the widespread downregulation of nonessential genes and metabolic processes to suppress energy expenditure and conserve endogenous fuel reserves. Such an extensive reorganization of cellular processes necessitates holistic changes to signaling pathways, enzyme activities, transcription/translation, and catabolism/anabolism. Not surprisingly, epigenetic controls have emerged as a valuable package of tools that allow organisms to adapt their metabolic processes in intricate and coordinated ways. Such epigenetic mechanisms include (a) modifications of DNA itself, (b) modifications on the histone proteins that wrap and structure nucleosomes, and (c) translational silencing by non-coding microRNA (miRNA) (Figure 1). Since epigenetic alterations alter gene expression without changing the DNA sequence itself, this makes them ideal for implementing rapid, transient changes in phenotype, which are needed to achieve both global control of MRD and short-term adaptive adjustments to changing environmental conditions. These are briefly described below.

DNA methylation involves the transfer of a methyl (-CH_3_) group from *S*-adenosylmethionine, to the 5′ carbon of a cytosine base to form 5-methylcytosine (5mC), a process catalyzed by the DNA methyltransferase (DNMT) family of enzymes. Such 5mC methylation patterns normally occur on CpG residues: a cytosine and a guanine nucleotide separated by a single phosphate group [10]. DNMT actions fall into two major categories: (1) maintenance methyltransferases, such as DNMT1, bind to hemi-methylated DNA to copy methylation patterns onto newly replicated DNA strands, and (2) de novo methyltransferases such as DNMT3A and DNMT3B place new methyl marks onto DNA [11]. A fourth family member, DNMT3L, is a non-canonical DNMT that possesses no catalytic activity, but instead forms complexes with DNMT3A and DNMT3B, as well as other epigenetic enzymes, to regulate their activity [12,13].

Histone modifications are major gatekeepers of gene expression. Histones are small positively charged proteins that make up the functional unit of chromatin, the nucleosome. The nucleosome consists of ~200 bp of DNA wrapped around a histone octamer, made of pairs of histones H2A, H2B, H3 and H4, which enable intense condensation of genetic material within cell nuclei. Chromatin remodeling is a characteristic effect of histone modification, providing for interconversion between transcriptionally permissive euchromatin and repressive heterochromatin [14]. Acetylation of lysine residues on histone tails (donated from acetyl-CoA) is tightly linked to gene activation via: (1) locally relaxing the tight electrostatic interactions of positively charged histone proteins with negatively charged DNA, (2) providing binding sites for bromodomain “reader” proteins to recruit transcriptional machinery, and (3) preventing the occupancy of silencing modifications, since multiple modifications cannot co-exist at the same lysine residue. Histone lysine methylation involves the addition of one, two or three methyl groups onto side chains of lysine residues, and most methyl-lysine marks have been shown to be strongly associated with transcriptional suppression [15,16].

MiRNA are short, single-stranded, noncoding RNA of 21–24 nt in length that bind to mature mRNA transcripts to suppress their translation by mediating either the degradation or sequestration of mRNA transcripts [17]. Since the discovery of the first miRNAs, *let-7* and *lin-4* in 1993, hundreds of miRNAs have been identified across animal and plant species, all of which exhibit very high conservation between species. Given such high conservation among vertebrates, and the possession of unique features that make miRNA ideal for implementing reversible, transient phenotypes, miRNAs represent a robust mode of post-transcriptional regulation that plays a crucial and dynamic role in allowing organisms to make rapid changes to mRNA translation in response to diverse signals including rapidly changing environmental conditions.

**Figure 2:**
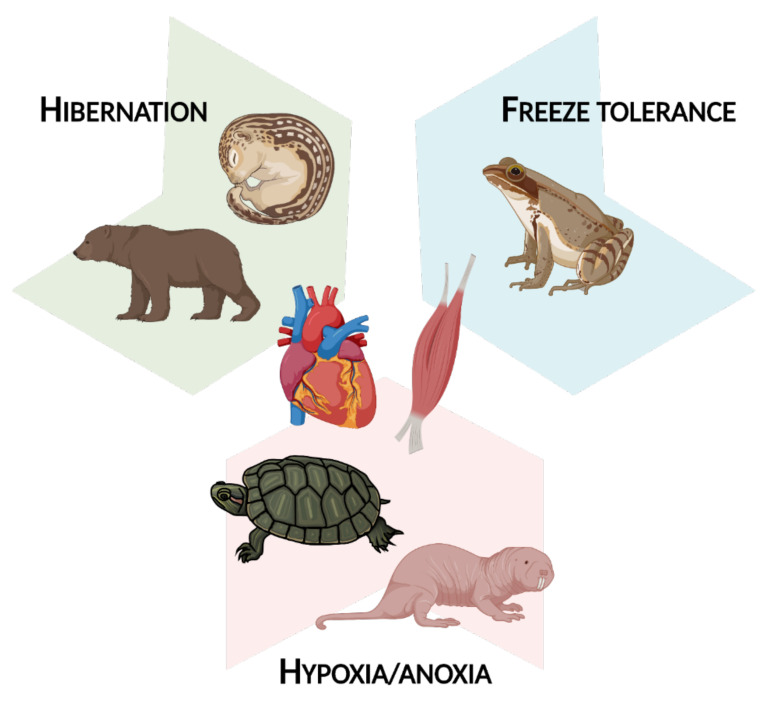
An overview of the animals and extreme environmental stresses covered in this review article. Figure created using BioRender.com (accessed on 7 October 2021).

There is undeniably wide variability in the functional changes that epigenetic modifications can bring about; the combinations of DNA modifications, histone modifications, and miRNA expression in response to different extreme environmental stresses can lead to a vast network of molecular responses. However, there are some common themes that epigenetic alterations help to orchestrate, and as such, this review article is split into three main sections. Part 2 concerns myoprotective and regenerative changes, most frequently seen in tandem with hibernation. Part 3 involves changes in cellular fuel usage during MRD, as observed in hibernation and under hypoxia/anoxia. Finally, Part 4 is about transcriptional and translational suppression, a common result of epigenetic changes in muscle tissue given that many animals are largely immobile in their winter-survival states. This occurs mostly during freeze tolerance and hypoxia/anoxia exposure. Overall, this review covers epigenetic changes in hibernation, freeze tolerance, and hypoxia/anoxia as it relates to muscle tissue (Figure 2).

## 2. Myoprotection and Regeneration

The aforementioned winter survival strategies of freeze tolerance, hibernation, and hypoxia/anoxia involve animals being largely nonambulatory for the duration of winter. This puts skeletal muscles at severe risk of atrophy from disuse, lack of exogenous nutrient intake, and waste export. Widespread muscle atrophy—defined as the wasting or loss of muscle tissue, a decline in force/power output, and the stimulation of muscle catabolism—can be catastrophic for an organism’s health from a physiological standpoint, and moreover, an animal that does not have sufficient muscle capacity to move is vulnerable to predation upon reversal of their respective winter survival strategy. Levels of skeletal muscle atrophy are drastically lower in hibernators than would be expected from a human in an equivalent bed-rest study, and indeed certain mechanisms are in place to counteract atrophy including urea nitrogen salvaging, which allows free nitrogen from muscle breakdown to be recycled into amino acids and thus serves a dual purpose of reconstituting muscle and preventing otherwise-hazardous ammonia toxicity [18]. We expect a facet of winter survival to be further dedicated to muscle maintenance and regeneration in the case of atrophy, which we detail in this section.

Myocyte enhancer factor 2, or MEF2, is a family of transcription factors that play direct roles in muscle plasticity and remodeling in response to physiological demands [19]. Four isoforms of MEF2 (MEF2A, -B, -C, -D) are known in mammals and all are regulated by reversible phosphorylation [20]. Due to the far-reaching and versatile abilities of MEF2-family transcription factors, it has been shown that the MEF2A signaling pathway is heavily affected during hibernation [21]. During hibernation, body temperature (T_b_) can fall to near ambient values (often 0–5 °C), and metabolic energy expenditure during hibernation can be reduced to as low as 1–5% of euthermic rates [22]. Prolonged torpor bouts in hibernating species alternate with short periods of arousal where T_b_ rises back to euthermic levels (near 37 °C) for several hours, during which restorative actions occur before animals sink into another bout of torpor. This regulation, and its reversal during rewarming, includes controls at transcriptional [23,24], translational [25], and post-translational levels [24,26] including activation of selected transcription factors, including MEF2A, which play pro-survival roles [21,27].

One such hibernator is the brown bear, *Ursus arctos*. *U. arctos* is a warm hibernator, defined by its T_b_ remaining near 37 °C for the full duration of hibernation, its huge body mass, making it essentially impossible for T_b_ to decrease significantly. While one might expect severe muscle wasting after 6–8 months of hibernation, studies have repeatedly shown minimal loss in skeletal muscle integrity when bears emerge from dens in the spring [28,29,30]. Epigenetic regulation is implicated in this ability. An investigation by Luu et al. [31] used RT-qPCR to analyze 36 miRNAs linked to MEF2A regulation in skeletal muscle samples from hibernating versus summer-active bears. Results showed that transcript levels of *mef2a* were upregulated during hibernation, with miRNAs under MEF2A control (miR-1b-5p, miR-133a-3p, and miR-206-3p) showing corresponding upregulation. Of these, miR-1 and miR-206 target *pax7* and *id2* genes, whose transcript levels are concurrently downregulated during hibernation. Other MEF2A-independent miRNAs with separate ties to muscle atrophy and regeneration were also quantified, with miR-23a-5p, miR-221-3p, and miR-31-5p being upregulated and miR-199a-5p and miR-223-5p downregulated during hibernation [31]. It is interesting to note that in human sarcopenia, miR-23a and miR-199 are two mitomiRs known to influence this disorder [32]. Together, this suggests that the activation of the MEF2A-mediated signaling pathway in hibernating bear skeletal muscle occurs through the upregulation of miR-1 and miR-206 and consequent downregulation of *pax7* and *id2* [31]. It should be noted that miR-27b, miR-29b, and miR-23a were also elevated in another hibernator during winter (little brown bat, *Myotis lucifugus*), and are also linked to muscle atrophy resistance, increased MEF2A activity, and decreased transcript levels of *mstn* that encodes myostatin, an inhibitor of muscle growth [33].

*M. lucifugus* undergoes a more “traditional” form of hibernation—a decrease in T_b_ to near ambient levels for prolonged periods but interspersed with brief arousal periods where T_b_ returns euthermic values for approximately a day. The Kornfeld et al. study found increases in eight miRNAs: miR-1a-1, miR-29b, miR-181b, miR-15a, miR-20a, miR-206, and miR-128-1 during torpor [33]. All of these miRNAs target muscle-specific factors that are implicated in muscle preservation during hibernation. Another well-studied hibernator is the 13-lined ground squirrel, *Ictidomys tridecemlineatus*. A study by Wu et al. [34] used RT-qPCR to quantify the levels of 117 miRNA across various time points of the hibernation-arousal cycle, and found a selection of 36 and 34 miRNAs in heart which changed during deep hibernation and interbout arousal, respectively, whereas 39 and 33 miRNAs changed at these two time points in skeletal muscle [34]. Of these, eight miRNAs were shared between heart and skeletal muscle during deep hibernation, one upregulated and seven downregulated. During interbout arousal, one shared miRNA was upregulated and two shared miRNAs were downregulated. Bioinformatic analysis of these miRNAs noted that miR-208, which was upregulated fivefold in heart tissue during arousal, is linked to myoprotective roles including cardiac arrhythmias and muscle remodeling [35]. However, in skeletal muscle, miR-208 was downregulated, highlighting tissue-specific roles for this, and possibly other, miRNAs that have yet to be fully elucidated. Another study of *I. tridecemlineatus* identified a subset of 17 novel miRNAs isolated via RT-qPCR [36]. In heart and skeletal muscle, these miRNAs were largely downregulated during hibernation and were proposed to have muscle remodeling roles, further functions of which have yet to be elucidated [36].

Hibernation is also an ability shared by selected primate species, including the grey mouse lemur, *Microcebus murinus*, of Madagascar. However, *M. murinus* and *U. arctos* differ in their hibernation strategies in a few key ways: *M. murinus* undergoes torpor, which is generally a daily reduction in metabolic rate during the inactive non-foraging hours, and is not limited to the winter months as it lasts for variable (and shorter) durations. In *M. murinus*, 16 miRNAs were identified with expression levels differing between control and torpid states; these miRNAs also have the potential to act as measurable biomarkers of torpor [37]. Moreover, this subset of miRNAs is involved in muscle-specific mechanisms and include miR-1 and miR-133, along with others that display unique torpor expression patterns. Readers should note that miR-1 is shared in common with hibernation in *U. arctos* [31]. In skeletal muscle of *M. murinus*, 20 miRNAs amid a group of 234 miRNAs assessed were significantly altered during torpor [37]. Eleven were significantly upregulated, and nine were significantly downregulated. Key members of the myomiR family were among those downregulated, and it was posited that suppression of this family may act to limit muscle proliferation during torpor, since this would be metabolically expensive and run counter to the general principle of MRD [38]. Moreover, two myomiRs (miR-1 and miR-133) directly target other potentially crucial processes including apoptosis, where reduced levels of miR-1 and/or miR-133 have been shown to favour survival [39].

## 3. Fuel Use: Glucose and Lipids

The switch to prolonged MRD during the winter necessitates a change in fuel usage, which manifests in a species- and stress-specific manner. In hibernation, fuel usage typically switches from carbohydrate metabolism to lipid fatty acid oxidation, as most hibernating species consume massive amounts of food in the late summer and autumn and stockpile it as white adipose for overwintering [40]. This transition includes changes in miRNAs; e.g., in brown bear *U. arctos*, levels of miR-27b-5p, 27a/b-3p, 29a-5p, 29a-3p, 29b/c-3p, 33-5p were upregulated in skeletal muscle during winter hibernation [31]. All of these are linked with glucose and lipid metabolism. The increases in miR-27, miR-29, and miR-33 suggest that these miRNAs may be potentially facilitating decreased glucose utilization, since miR-27 has been linked to decreasing glucose uptake in rat muscles via targeting of *glut4* that encodes glucose transporter 4 [41]. GLUT4 is the primary glucose transporter responsible for insulin-stimulated uptake of glucose into muscle cells, and its activity can be tailored for increased or decreased glucose uptake in response to environmental stress [42]. In contrast, miR-33 targets *prkaa1* (which encodes a catalytic subunit of the AMP-activated protein kinase, AMPK). AMPK primarily increases glucose uptake and promotes fatty acid oxidation by phosphorylating ACC and decreasing malonyl-CoA production [43], but *prkaa1* transcript levels did not change in hibernation [31]. Overall, increases in miR-27, miR-29, and miR-33 in *U. arctos* skeletal muscle may suggest that both glucose uptake and fatty acid oxidation are suppressed in hibernating bears, signifying a miRNA-mediated switch in fuel usage during the winter [31].

Continuing on the theme of MRD, we turn to a new extreme environmental stress that has not yet been explored in-depth with respect to miRNA involvement. Naked mole rats, *Heterocephalus glaber*, are commonly exposed to low levels of oxygen in their burrows, creating hypoxic conditions—defined as low (suboptimal) availability of oxygen—where O_2_ levels can be as low as 3–10% [44,45]. Many animals experience hypoxia/anoxia as a result of their environmental conditions, particularly among various aquatic species such as those that undergo breath-hold diving, including red-eared slider turtle *Trachemys scripta elegans*. These species will be addressed later in this review. The hallmarks of MRD are maintained in species that survive hypoxia/anoxia—metabolic rate is strongly reduced and nonessential or energy-expensive processes are downregulated. Energy usage is reprioritized to support survival mechanisms including antioxidant defenses, anti-apoptotic mechanisms, and anaerobic glycolysis. A study by Hadj-Moussa et al. [46] examined hypoxia effects on *H. glaber* and measured the levels of miRNAs previously implicated in oxygen responsiveness. Of 55 miRNAs measured, 10 were differentially regulated in *H. glaber* skeletal muscle with miR-101a-3p, miR-124-3p, and miR-199a-5p upregulated during hypoxia whereas miR-137-3p, miR-200c-3p, miR-214-3p, miR-223-3p, miR-296-5p, miR-325-3p, and miR-503-5p were downregulated [46]. All these miRNAs are related to AMPK control, providing an intriguing link to *U. arctos* hibernation and *prkaa1* as described in [31]. AMPK and its role in hypoxia has been well-characterized in a variety of tissue types including skeletal muscle [47,48] and cardiomyocytes [49], so it makes sense that we would observe epigenetic control of AMPK in *H. glaber*. Of the three upregulated miRNAs in hypoxia, miR-124 overexpression negatively regulates cell growth and glucose consumption in cancer cells [50], in line with AMPK downregulation. Additionally, miR-124 has been observed to increase phosphorylated-AMPK levels in brain tissues, suggesting a similar role may exist in skeletal muscle [51]. Upregulated levels of miR-101a and miR-199a are associated with reductions in AMPK signaling and promotion of fatty acid synthesis [52], and miR-101a can both directly and indirectly inactivate AMPK [53,54]. The seven downregulated miRNAs during hypoxia are also implicated in AMPK signaling in various downstream roles in combating oxidative stress, autophagy and apoptosis during hypoxia, or following anoxia/reoxygenation. Returning to fuel consumption and the overall roll of AMPK in skeletal muscle, it is known that *H. glaber* mobilizes glucose from the liver to the blood during severe hypoxia [55]. Since miRNA expression in skeletal muscle suggests that AMPK is downregulated, thereby promoting fatty acid metabolism and a shift away from glucose uptake, an explanation may be that glucose is reprioritized for the brain during hypoxia, an organ very sensitive to ATP limitation and this can be mitigated, in part, by reducing glucose uptake and use by muscle cells.

AMPK has another role of promoting mitochondrial biogenesis, thus enhancing cellular abilities to provide energy substrates [56]. Downregulation of AMPK via miRNAs as highlighted in this subsection may cause a corresponding decrease in mitochondrial processes, which could be advanced by a specific family of miRNAs known as mitomiRs. This miRNA family was originally derived from research into humanin and its conserved role in oxidative stress cytoprotection, and now they are hypothesized to influence mitochondrial behavior during extreme environmental stresses, given they are already linked with mitochondrial functions ranging from energy metabolism to apoptosis [57].

The functions and regulatory roles of mitomiRs in mitochondrial energy metabolism have begun to be elucidated [58]. For example, mitomiR-181c has been shown to suppress the levels of mitochondrial COX-1, a subunit of complex IV of the electron transport chain 12, while mitomiR-378a regulates glucose homeostasis via inhibiting mitochondrial ATP6. Other studies have shown mitomiRs involvement in cardiovascular diseases and mitochondrial dysfunction. One study demonstrated that levels of mitomiR-696, mitomiR-532 and mitomiR-690 rose during the initial stages of heart failure with this upregulation linked to changes in mitochondrial energetics [59], while mitomiR-378a has been shown to inhibit lactate dehydrogenase and induce apoptosis in cardiomyocytes [60]. Other mitomiRs such as let-7b, miR-146a, miR-19b, miR-34a and miR221 all have associations with cell senescence [61]. Overall, many studies have established the role of mitomiRs in cardiovascular processes either by regulating glucose metabolism, mitochondrial energetics or genes in mitochondrial metabolism suggesting a possible promise that they hold for future insights into survival of extreme environmental stresses.

## 4. Transcriptional and Translational Suppression

A critical theme of MRD is the notion of global downregulation—nonessential genes and processes are shut down or off to preserve limited cellular energy for pro-survival processes. Transcriptional suppression is a holistic definition for preventing transcription of the DNA strand, which can be enacted through various epigenetic mechanisms detailed throughout this review, and translational repression is the limitation of translating mRNA transcripts into protein. DNA methylation is widely associated with transcriptional suppression, since the addition of methyl group(s), provided by *S-*adenosylmethionine, onto cytosine residues interferes with transcription factor binding and prevents transcription. One function of histone methylation tightens nucleosomes and prevents access to chromatin, and miRNA acts on a post-transcriptional level by preventing translation of mRNA transcripts. The first two sections of this review have focused on specific cellular processes affected through epigenetic alterations, but many of the suppressive features of epigenetics including DNA methylation and miRNA action contribute to MRD on a broad sense. This section elucidates studies of this nature.

DNA methylation has been examined in heart and skeletal muscle during hibernation of *I. tridecemlineatus* [62]. In heart, DNMT1, DNMT2, and DNMT3L were upregulated during deep torpor, whereas DNMT3A remained steady during hibernation but decreasing during interbout arousal [62]. DNMT enzyme activity increased during hibernation, but there was no change in 5mC levels. In skeletal muscle, DNMT3L was the only methyltransferase to change with an upregulation during interbout arousal; however, DNMT enzyme activity was drastically decreased across all time points and 5mC levels increased during interbout arousal [62]. This aligns with data from a study by [63], which showed that *dnmt1* and *dnmt3b* transcript levels in skeletal muscle did not change at any point during hibernation. Given the differential nature of these responses, it was hypothesized that DNA methylation plays roles in a more transcription-factor and gene-dependent manner which provides a more gene-specific function rather than a broad-spectrum approach. For example, [63] showed a decrease in *mef2c* transcript levels at all points during hibernation, perhaps tying this into a myoprotection and regeneration role (as discussed in Part 1). DNMT3L function as a cofactor rather than a catalytic member of the family also allows for other epigenetic functions to be at play, rather than looking at DNA methylation in isolation. For example, miR-24 was downregulated in both heart and skeletal muscle of 13-lined ground squirrels, which strongly suggests suppression of cellular growth and proliferation during hibernation, in line with global MRD [64].

We can also look back to the study by [37] that measured changes in miRNAs in skeletal muscle of the mouse lemur, *M. murinus*, during daily torpor. It has already been noted that several miRNAs play myoprotective roles in this primate, including miR-1 and miR-133. Of the upregulated miRNAs, many others were related to cell growth including miR-2478 and miR-889 [37]. These target transcripts of *tgfβ1*, which encodes the TGFβ1a receptor that is linked with the BMP family of proteins to form the SMAD signaling pathway. SMADs play major roles in cell proliferation and differentiation [65]. Downregulation of signaling pathways have been associated with metabolic rate depression in many species and in response to multiple stresses, likely acting to suppress energy-expensive processes during torpor/hibernation [66,67,68]. Research on the torpor-capable marsupial *Dromiciops gliroides* measured the levels of 85 miRNAs in skeletal muscle, showing significant upregulation of seven miRNAs and downregulation of four miRNAs [69]. As predicted by bioinformatic analysis, these miRNAs primarily targeted mRNA species associated with signaling pathways, including the ErbB and mTOR pathways. Since signaling pathways are metabolically costly to maintain, miRNA action in *D. gliroides* muscle appears to also be leading to translational suppression and maintenance of MRD.

Transcriptional suppression is also a hallmark of freeze tolerance, most notably exemplified in studies of the wood frog, *Rana sylvatica*. Freeze tolerance is a survival strategy that has been documented for many species living in seasonally cold environments, including several species of vertebrates (frogs, salamanders, hatchling turtles) and many invertebrates (insects, molluscs) [4,9,70]. Freeze-tolerant animals allow ice formation within extracellular and extra-organ cavities (but not inside cells), together with the cessation of heartbeat, breathing, and movement. Extracellular ice buildup draws water out of cells, creating a hyperosmotic intracellular environment and extreme cell shrinkage that is combated by the synthesis of low molecular weight cryoprotectants that are flooded throughout the body and taken up by cells to combat cell water loss. These cryoprotectants include a variety of small molecules: polyhydric alcohols (e.g., glycerol, sorbitol), sugars (e.g., glucose, trehalose) and small nitrogenous compounds (e.g., urea). Freeze-tolerant species can often endure the conversion of ~65% of total body water into ice and freezing halts blood flow imposing hypoxia/anoxia as oxygen is depleted [4]. Gas exchange and waste export is halted, and skeletal muscle atrophy is a risk from prolonged lack of use.

MicroRNA has again been identified as a crucial regulator of MRD in freeze tolerance, acting as a transcriptional suppressor. Studies of miRNA regulation over the freeze/thaw cycle in *R. sylvatica* have analyzed heart and skeletal muscle responses, in which qPCR was used to quantify levels of 53 miRNAs in these tissues [71]. Of the miRNAs tested in heart, only one miRNA was upregulated, whereas four were downregulated during freezing, although a larger subset of 20 was downregulated after 8 h thawing [71]. The widespread downregulation of miRNAs during thawing may signify that many cellular processes need to be reactivated after thawing to cope with probable accumulated damage from freezing. Indeed, selected miRNAs from the group that was analyzed are known to play roles in heart function, such as miR-145 and miR-208, and are known to be overexpressed in various heart diseases [72]. Skeletal muscle showed an alternate trend, with 16 miRNAs upregulated and one downregulated during freezing, as well as six remaining upregulated after thawing [71]. The miRNAs affected in skeletal muscle targeted genes associated with the cell cycle and apoptosis, suggesting that these processes are suppressed during freezing and remain this way throughout the thaw. Bioinformatic prediction of pathways affected by these miRNAs yielded targets including actin cytoskeleton, PI3K/Akt, and MAPK signaling as being disproportionately affected by miRNAs [71]. A focus on intracellular signal transduction has also been noted previously in wood frogs during freezing, suggesting a more global theme for the functions of miRNA in regulating signaling pathways during freezing. For example, signaling networks including PI3K/Akt and mTOR have been implicated to be under miRNA regulatory control during freezing in wood frog brain, and the Akt signaling pathway may be freezing-dependent on miRNA regulation in wood frog liver [73,74].

Another aspect of transcriptional control is direct inhibition of DNA transcription via DNA methylation. The authors of [63] examined *R. sylvatica* skeletal muscle, and found that both DNMT1 and DNMT3L were upregulated in response to 24 h freezing, whereas DNMT3A/3B remained unchanged along with overall DNMT enzyme activity. However, global 5mC levels were upregulated, demonstrating that there was a measurable increase in genomic methylation during freezing [75]. Following an 8-hour thaw, DNMT1 and 5mC levels returned to baseline, whereas DNMT3L increased even further [75]. Interestingly, total DNMT activity was strongly suppressed after 8 h of thawing. DNMT1 is a maintenance methyltransferase, responsible for methylating hemi-methylated DNA following a round of DNA replication [11,76]. DNMT3L is not a canonical member of the DNMT family and does not possess any catalytic activity of its own but acts as a cofactor to allow greater affinity for DNA and, therefore, more efficient function. Taken collectively, this appears to be a counterintuitive result, given that skeletal muscle is one of the first tissues to freeze and has a low metabolic activity during freezing. However, a potential role for DNMT1 involves its noncanonical ability to interact with histone-modifying genes including EZH2, which is part of a complex PCR2 mediating repressive trimethylations on H3 lysines [77]. DNMT3L is also known to interact with histone-related genes including HDAC1 [78,79], so it is possible that elevated DNMT1 and DNMT3L in skeletal muscle could be performing noncatalytic roles by influencing histone modifications.

Transcriptional suppression is also seen during MRD in situations of hypoxia/anoxia stress. The red-eared slider turtle, *T. s. elegans*, is a well-studied model of anoxia tolerance both during diving excursions in warm seasons and prolonged periods (up to 4–5 months) of continuous submergence in cold water during the winter. These turtles live in ponds, lakes or streams that typically become ice-locked during the winter, preventing turtles from rising to the surface to breathe. Although some turtle species can take up oxygen over buccal or cloacal membranes, sliders and closely related painted turtles (*Chrysemys*) do not [80]. Instead, they sustain life with a combination of strong metabolic rate depression (to as low as 10% of aerobic values), huge reserves of glycogen in liver, and lactic acid buffering/storage in the shell. A study by Wijenayake and Storey [81] measured DNMT protein levels in white muscle, heart and liver of *T. s. elegans.* In white muscle, DNMT3A and DNMT3B protein levels increased by three-fold after just 5 h of anoxic submergence. After 20 h of anoxia, DNMT3A had returned to control levels, but DNMT3B remained high [81]. Total DNMT activity increased in both 5 h and 20 h anoxia, and global 5mC methylation rose significantly in the 5 h anoxia condition but declined slightly (but remained higher than controls) after 20 h anoxia [81]. In heart, DNMT3A was also elevated after 5 h anoxia and DNMT3B had increased strongly after 20 h anoxia. Total DNMT activity and global 5mC levels, however, remained unchanged. These results suggest that DNMT regulation is not derived solely from increased or decreased protein synthesis, given that there was no change in either activity or genome methylation. There may be another mechanism that affects DNMTs and supports more efficient methyltransferase activity, without necessarily leading to quantitative increases or decreases measurable in the study. One possibility is that global 5mC levels were measured on a whole-cell level, and it is possible that changes in methylation occur in a gene-specific manner that does not result in significant changes to genome-wide methylation. Global 5mC levels reflected this increase in activity in white muscle, whereas 5mC levels in heart remained unchanged after 5 h anoxia (corresponding to the unchanged DNMT activity) and remained consistent during 20 h anoxia even though DNMT activity was increased at this time point.

As with the other species examined in this review, miRNA regulation has been analyzed in *T.s. elegans* to elucidate its contribution to post-transcriptional suppression during anoxia. Two studies have best exemplified this. Biggar and Storey [82] examined transcript and protein levels of cyclin D1, a crucial regulator of the G_1_ phase that initiates the cell cycle, as well as miRNAs that target cyclin D1. Under anoxia, *cyclin D1* transcript levels did not change in liver or kidney and neither did phosphorylation of cyclin D1 at Thr286, but cyclin D1 protein decreased significantly in both organs. This implicated miRNA action in the suppression of cyclin D1 levels under anoxia and was supported by increased levels of miR-16-1 and miR-15a under anoxia and the presence of conserved binding sites for these two microRNAs in the 3′ UTR of the *cyclin D1* gene. A further study examined the responses of a range of miRNAs under low temperature and anoxic conditions [83]. The 2017 study further explored the roles of miRNAs in turtle anoxia tolerance using RT-qPCR and found that miR-1a, miR-107, miR-20a, miR-21, and miR-29b were upregulated after both 5 h and 20 h anoxia exposure, whereas miR-148a was elevated only after 20 h anoxia [83]. Conversely, miR-17 was downregulated in both 5 h and 20 h anoxia, the only miRNA measured to do so. Of note were miR-21 and miR-20a; both of these miRNAs are hypothesized to fulfill anti-apoptotic roles and suppress cellular proliferation in *T.s. elegans*, continuing the theme of post-transcriptional suppression during anoxia. It is interesting to note that bioinformatic analysis predicted that lower temperatures would lead to more and stronger miRNA:mRNA interactions, indicative of stronger suppression of mRNA translation in the cold and opening and an intriguing new area for future research.

## 5. Future Directions

It should be noted that histone modifications were not covered in this article. The existing research regarding histone modifications in muscle during winter is in its infancy, and it has been difficult to establish definitive functional links arising from changes in histone methylation/acetylation. Therefore, the existing literature was not robust enough to warrant inclusion in this article. Preliminary studies including [84], [85], and [86] provide introductions into histone modifications in *I. tridecemlineatus*, *R. sylvatica*, and *T.s. elegans*, respectively. Future research will no doubt elucidate the functions and full breadth of histone modifications in muscle during winter survival, and how they integrate into other epigenetic mechanisms, as detailed herein.

A finding in [74,83] also merits further note. In a bioinformatic prediction comparing miRNA:mRNA interactions at 5 and 37 °C, it was found that the lower temperature allowed for approximately twice as many interactions as compared to the higher temperature. Given that many winter survival strategies are inextricably linked with a low or subzero T_b_, it will be critical to incorporate this thermodynamic factor into future research to understand the full extent of miRNA regulation of mRNA translation in mediating metabolic adaptations for low temperature survival.

## 6. Conclusions

In this review article, we laid out the recent advances in epigenetics research and how they tie into survival of extreme environmental stresses induced by winter conditions. Across all stresses, epigenetic influences fall under the three major themes: (1) myoprotection and regeneration, (2) altered fuel use between glucose and lipids, and (3) holistic transcriptional and translational suppression. The involvement of miR-1 was observed in both hibernation (in two mammalian species) and anoxia (in *T.s. elegans*), signifying that this miRNA may have major significance in muscle metabolism over the winter. The AMPK family that mediates glucose and lipid catabolism is also affected by epigenetic controls, as denoted in hypoxic *H. glaber* and hibernating *U. arctos*. Finally, downregulation of signaling pathways including AMPK, cell cycle, MAPK, and SMAD signaling are affected via both miRNA control and DNA methylation across many species and winter-survival strategies. Overall, the maintenance of muscle tissue during winter is of critical importance, and research has shown that epigenetic mechanisms contribute to this role by exerting powerful regulatory control over key aspects of winter survival, bridging species gaps and highlighting shared mechanisms which can pave the way for future research.

## References

[1] Storey K.B., Storey J.M., Makowski G. (2010). Metabolic Rate Depression. The Biochemistry of Mammalian Hibernation. Advances in Clinical Chemistry.

[2] Storey K.B., Storey J.M. (2012). Aestivation: Signaling and Hypometabolism. J. Exp. Biol..

[3] Krivoruchko A., Storey K.B. (2015). Turtle Anoxia Tolerance: Biochemistry and Gene Regulation. Biochim. Et Biophys. Acta.

[4] Storey K.B., Storey J.M. (2017). Molecular Physiology of Freeze Tolerance in Vertebrates. Physiol. Rev..

[5] Ring R.A. (1982). Freezing-Tolerant Insects with Low Supercooling Points. Comp. Biochem. Physiol..

[6] Thomashow M.F. (1999). Plant Cold Acclimation: Freezing Tolerance Genes and Regulatory Mechanisms. Annu. Rev. Plant Physiol. Plant Mol. Biol..

[7] Costanzo J.P., Lee R.E., Ultsch G.R. (2008). Physiological Ecology of Overwintering in Hatchling Turtles. J. Exp. Zool. Part A Ecol. Genet. Physiol..

[8] Holmstrup M. (2014). The Ins and Outs of Water Dynamics in Cold Tolerant Soil Invertebrates. J. Therm. Biol..

[9] Murphy D.J. (1983). Freezing Resistance in Intertidal Invertebrates. Annu. Rev. Physiol..

[10] Bird A.P. (1986). CpG-Rich Islands and the Function of DNA Methylation. Nature.

[11] Lyko F. (2018). The DNA Methyltransferase Family: A Versatile Toolkit for Epigenetic Regulation. Nat. Rev. Genet..

[12] Chédin F., Lieber M.R., Hsieh C.L. (2002). The DNA Methyltransferase-like Protein DNMT3L Stimulates de Novo Methylation by DNMT3A. Proc. Natl. Acad. Sci. USA.

[13] Suetake I., Shinozaki F., Miyagawa J., Takeshima H., Tajima S. (2004). DNMT3L Stimulates the DNA Methylation Activity of DNMT3A and DNMT3B through a Direct Interaction. J. Biol. Chem..

[14] Kouzarides T. (2007). Chromatin Modifications and Their Function. Cell.

[15] Barski A., Cuddapah S., Cui K., Roh T.Y., Schones D.E., Wang Z., Wei G., Chepelev I., Zhao K. (2007). High-Resolution Profiling of Histone Methylations in the Human Genome. Cell.

[16] Mikkelsen T.S., Ku M., Jaffe D.B., Issac B., Lieberman E., Giannoukos G., Alvarez P., Brockman W., Kim T.K., Koche R.P. (2007). Genome-Wide Maps of Chromatin State in Pluripotent and Lineage-Committed Cells. Nature.

[17] Bartel D.P. (2004). MicroRNAs: Genomics, Biogenesis, Mechanism, and Function. Cell.

[18] Rice S.A., Ten Have G.A.M., Reisz J.A., Gehrke S., Stefanoni D., Frare C., Barati Z., Coker R.H., D’Alessandro A., Deutz N.E.P. (2020). Nitrogen Recycling Buffers against Ammonia Toxicity from Skeletal Muscle Breakdown in Hibernating Arctic Ground Squirrels. Nat. Metab..

[19] Tessier S.N., Storey K.B. (2016). Lessons from Mammalian Hibernators: Molecular Insights into Striated Muscle Plasticity and Remodeling. Biomol. Concepts.

[20] Black B.L., Olson E.N. (1998). Transcriptional Control of Muscle Development by Myocyte Enhancer Factor-2 (MEF2) Proteins. Annu. Rev. Cell Dev. Biol..

[21] Tessier S.N., Storey K.B. (2010). Expression of Myocyte Enhancer Factor-2 and Downstream Genes in Ground Squirrel Skeletal Muscle during Hibernation. Mol. Cell. Biochem..

[22] Carey H.V., Andrews M.T., Martin S.L. (2003). Mammalian Hibernation: Cellular and Molecular Responses to Depressed Metabolism and Low Temperature. Physiol. Rev..

[23] Srere H.K., Wang L.C.H., Martin S.L. (1992). Central Role for Differential Gene Expression in Mammalian Hibernation. Proc. Natl. Acad. Sci. USA.

[24] Morin P., Storey K.B. (2006). Evidence for a Reduced Transcriptional State during Hibernation in Ground Squirrels. Cryobiology.

[25] Wu C.W., Storey K.B. (2012). Regulation of the MTOR Signaling Network in Hibernating Thirteen-Lined Ground Squirrels. J. Exp. Biol..

[26] Abnous K., Dieni C.A., Storey K.B. (2012). Suppression of MAPKAPK2 during Mammalian Hibernation. Cryobiology.

[27] Tessier S.N., Storey K.B. (2012). Myocyte Enhancer Factor-2 and Cardiac Muscle Gene Expression during Hibernation in Thirteen-Lined Ground Squirrels. Gene.

[28] Hershey J.D., Robbins C.T., Nelson O.L., Lin D.C. (2008). Minimal Seasonal Alterations in the Skeletal Muscle of Captive Brown Bears. Physiol. Biochem. Zool..

[29] Harlow H.J., Lohuis T., Beck T.D.I., Iaizzo P.A. (2001). Muscle Strength in Overwintering Bears. Nature.

[30] Tinker D.B., Harlow H.J., Beck T.D.I. (2015). Protein Use and Muscle-fiber Changes in Free-ranging, Hibernating Black Bears. Physiol. Zool..

[31] Luu B.E., Lefai E., Giroud S., Swenson J.E., Chazarin B., Gauquelin-Koch G., Arnemo J.M., Evans A.L., Bertile F., Storey K.B. (2020). MicroRNAs Facilitate Skeletal Muscle Maintenance and Metabolic Suppression in Hibernating Brown Bears. J. Cell. Physiol..

[32] Cannataro R., Carbone L., Petro J.L., Cione E., Vargas S., Angulo H., Forero D.A., Odriozola-Martínez A., Kreider R.B., Bonilla D.A. (2021). Sarcopenia: Etiology, Nutritional Approaches, and MiRNAs. Int. J. Mol. Sci..

[33] Kornfeld S.F., Biggar K.K., Storey K.B. (2012). Differential Expression of Mature MicroRNAs Involved in Muscle Maintenance of Hibernating Little Brown Bats, *Myotis Lucifugus*: A Model of Muscle Atrophy Resistance. Genom. Proteom. Bioinform..

[34] Wu C.W., Biggar K.K., Luu B.E., Szereszewski K.E., Storey K.B. (2016). Analysis of MicroRNA Expression during the Torpor-Arousal Cycle of a Mammalian Hibernator, the 13-Lined Ground Squirrel. Physiol. Genom..

[35] Oliveira-Carvalho V., Carvalho V.O., Bocchi E.A. (2013). The Emerging Role of MiR-208a in the Heart. DNA Cell Biol..

[36] Luu B.E., Biggar K.K., Wu C.-W., Storey K.B. (2016). Torpor-Responsive Expression of Novel MicroRNA Regulating Metabolism and Other Cellular Pathways in the Thirteen-Lined Ground Squirrel, Ictidomys Tridecemlineatus. FEBS Lett..

[37] Hadj-Moussa H., Zhang J., Pifferi F., Perret M., Storey K.B. (2020). Profiling Torpor-Responsive MicroRNAs in Muscles of the Hibernating Primate *Microcebus Murinus*. Biochim. Biophys. Acta.

[38] McCarthy J.J. (2011). The MyomiR Network in Skeletal Muscle Plasticity. Exerc. Sport Sci. Rev..

[39] Xu C., Lu Y., Pan Z., Chu W., Luo X., Lin H., Xiao J., Shan H., Wang Z., Yang B. (2007). The Muscle-Specific MicroRNAs MiR-1 and MiR-133 Produce Opposing Effects on Apoptosis by Targeting HSP60, HSP70 and Caspase-9 in Cardiomyocytes. J. Cell Sci..

[40] Storey K.B. (2010). Out Cold: Biochemical Regulation of Mammalian Hibernation—A Mini-Review. Gerontology.

[41] Zhou T., Meng X., Che H., Shen N., Xiao D., Song X., Liang M., Fu X., Ju J., Li Y. (2016). Regulation of Insulin Resistance by Multiple MiRNAs via Targeting the GLUT4 Signalling Pathway. Cell. Physiol. Biochem..

[42] Vargas E., Podder V., Sepulveda M.A.C. (2021). Physiology, Glucose Transporter Type 4. StatPearls.

[43] Ke R., Xu Q., Li C., Luo L., Huang D. (2018). Mechanisms of AMPK in the Maintenance of ATP Balance during Energy Metabolism. Cell Biol. Int..

[44] Chung D., Dzal Y.A., Seow A., Milsom W.K., Pamenter M.E. (2016). Naked Mole Rats Exhibit Metabolic but Not Ventilatory Plasticity Following Chronic Sustained Hypoxia. Proc. R. Soc. B.

[45] Edrey Y.H., Hanes M., Pinto M., Mele J., Buffenstein R. (2011). Successful Aging and Sustained Good Health in the Naked Mole Rat: A Long-Lived Mammalian Model for Biogerontology and Biomedical Research. ILAR J..

[46] Hadj-Moussa H., Chiasson S., Cheng H., Eaton L., Storey K.B., Pamenter M.E. (2021). MicroRNA-Mediated Inhibition of AMPK Coordinates Tissue-Specific Downregulation of Skeletal Muscle Metabolism in Hypoxic Naked Mole-Rats. J. Exp. Biol..

[47] Mu J., Brozinick J.T., Valladares O., Bucan M., Birnbaum M.J. (2001). A Role for AMP-Activated Protein Kinase in Contraction- and Hypoxia-Regulated Glucose Transport in Skeletal Muscle. Mol. Cell.

[48] Siques P., Brito J., Flores K., Ordenes S., Arriaza K., Pena E., León-Velarde F., de Pablo Á.L.L., Gonzalez M.C., Arribas S. (2018). Long-Term Chronic Intermittent Hypobaric Hypoxia Induces Glucose Transporter (GLUT4) Translocation Through AMP-Activated Protein Kinase (AMPK) in the Soleus Muscle in Lean Rats. Front. Physiol..

[49] Marsin A.S., Bertrand L., Rider M.H., Deprez J., Beauloye C., Vincent M.F., van den Berghe G., Carling D., Hue L. (2000). Phosphorylation and Activation of Heart PFK-2 by AMPK Has a Role in the Stimulation of Glycolysis during Ischaemia. Curr. Biol..

[50] Zhao X., Lu C., Chu W., Zhang B., Zhen Q., Wang R., Zhang Y., Li Z., Lv B., Li H. (2017). MicroRNA-124 Suppresses Proliferation and Glycolysis in Non-Small Cell Lung Cancer Cells by Targeting AKT-GLUT1/HKII. Tumor Biol..

[51] Gong X., Wang H., Ye Y., Shu Y., Deng Y., He X., Lu G., Zhang S. (2016). MiR-124 Regulates Cell Apoptosis and Autophagy in Dopaminergic Neurons and Protects Them by Regulating AMPK/MTOR Pathway in Parkinson’s Disease. Am. J. Transl. Res..

[52] Li Y., Luan Y., Li J., Song H., Li Y., Qi H., Sun B., Zhang P., Wu X., Liu X. (2020). Exosomal MiR-199a-5p Promotes Hepatic Lipid Accumulation by Modulating MST1 Expression and Fatty Acid Metabolism. Hepatol. Int..

[53] Li Y., Wang S., Gao X., Zhao Y., Li Y., Yang B., Zhang N., Ma L. (2018). Octreotide Alleviates Autophagy by Up-Regulation of MicroRNA-101 in Intestinal Epithelial Cell Line Caco-2. Cell. Physiol. Biochem..

[54] Liu P., Ye F., Xie X., Li X., Tang H., Li S., Huang X., Song C., Wei W., Xie X. (2016). Mir-101-3p Is a Key Regulator of Tumor Metabolism in Triple Negative Breast Cancer Targeting AMPK. Oncotarget.

[55] Pamenter M.E., Dzal Y.A., Thompson W.A., Milsom W.K. (2019). Do Naked Mole Rats Accumulate a Metabolic Acidosis or an Oxygen Debt in Severe Hypoxia?. J. Exp. Biol..

[56] Jornayvaz F.R., Shulman G.I. (2010). Regulation of Mitochondrial Biogenesis. Essays Biochem..

[57] Borralho P.M., Rodrigues C.M.P., Steer C.J. (2015). MicroRNAs in Mitochondria: An Unexplored Niche. Adv. Exp. Med. Biol..

[58] Das S., Ferlito M., Kent O.A., Fox-Talbot K., Wang R., Liu D., Raghavachari N., Yang Y., Wheelan S.J., Murphy E. (2012). Nuclear MiRNA Regulates the Mitochondrial Genome in the Heart. Circ. Res..

[59] Wang X., Song C., Zhou X., Han X., Li J., Wang Z., Shang H., Liu Y., Cao H. (2017). Mitochondria Associated MicroRNA Expression Profiling of Heart Failure. BioMed Res. Int..

[60] Machado I.F., Teodoro J.S., Palmeira C.M., Rolo A.P. (2020). MiR-378a: A New Emerging MicroRNA in Metabolism. Cell. Mol. Life Sci..

[61] Rippo M.R., Olivieri F., Monsurrò V., Prattichizzo F., Albertini M.C., Procopio A.D. (2014). MitomiRs in Human Inflamm-Aging: A Hypothesis Involving MiR-181a, MiR-34a and MiR-146a. Exp. Gerontol..

[62] Tessier S.N., Ingelson-Filpula W.A., Storey K.B. (2021). Epigenetic Regulation by DNA Methyltransferases during Torpor in the Thirteen-Lined Ground Squirrel *Ictidomys Tridecemlineatus*. Mol. Cell. Biochem..

[63] Alvarado S., Mak T., Liu S., Storey K.B., Szyf M. (2015). Dynamic Changes in Global and Gene-Specific DNA Methylation during Hibernation in Adult Thirteen-Lined Ground Squirrels, *Ictidomys Tridecemlineatus*. J. Exp. Biol..

[64] Lang-Ouellette D., Morin P.J. (2014). Differential Expression of MiRNAs with Metabolic Implications in Hibernating Thirteen-Lined Ground Squirrels, *Ictidomys Tridecemlineatus*. Mol. Cell. Biochem..

[65] Derynck R., Feng X. (1997). TGF-Beta Receptor Signaling. Biochim. Biophys. Acta.

[66] Li Z., Lan X., Han R., Wang J., Huang Y., Sun J., Guo W., Chen H. (2017). MiR-2478 Inhibits TGFβ1 Expression by Targeting the Transcriptional Activation Region Downstream of the TGFβ1 Promoter in Dairy Goats. Sci. Rep..

[67] Biggar K.K., Storey K.B. (2018). Functional Impact of MicroRNA Regulation in Models of Extreme Stress Adaptation. J. Mol. Cell Biol..

[68] Logan S.M., Wu C.W., Storey K.B. (2019). The Squirrel with the Lagging EIF2: Global Suppression of Protein Synthesis during Torpor. Comp. Biochem. Physiol. Part A Mol. Integr. Physiol..

[69] Hadj-Moussa H., Moggridge J.A., Luu B.E., Quintero-Galvis J.F., Gaitán-Espitia J.D., Nespolo R.F., Storey K.B. (2016). The Hibernating South American Marsupial, *Dromiciops Gliroides*, Displays Torpor-Sensitive MicroRNA Expression Patterns. Sci. Rep..

[70] Storey K.B., Storey J.M. (1988). Freeze Tolerance in Animals. Physiol. Rev..

[71] Bansal S., Luu B.E., Storey K.B. (2016). MicroRNA Regulation in Heart and Skeletal Muscle over the Freeze–Thaw Cycle in the Freeze Tolerant Wood Frog. J. Comp. Physiol. B Biochem. Syst. Environ. Physiol..

[72] Cooley N., Cowley M.J., Lin R.C.Y., Marasco S., Wong C., Kaye D.M., Dart A.M., Woodcock E.A. (2012). Influence of Atrial Fibrillation on MicroRNA Expression Profiles in Left and Right Atria from Patients with Valvular Heart Disease. Physiol. Genom..

[73] Zhang J., Storey K.B. (2013). Akt Signaling and Freezing Survival in the Wood Frog, *Rana Sylvatica*. Biochim. Et Biophys. Acta Gen. Subj..

[74] Hadj-Moussa H., Storey K.B. (2018). Micromanaging Freeze Tolerance: The Biogenesis and Regulation of Neuroprotective MicroRNAs in Frozen Brains. Cell. Mol. Life Sci..

[75] Zhang J., Hawkins L.J., Storey K.B. (2019). DNA Methylation and Regulation of DNA Methyltransferases in a Freeze Tolerant Vertebrate. Biochem. Cell Biol..

[76] Bashtrykov P., Jankevicius G., Smarandache A., Jurkowska R.Z., Ragozin S., Jeltsch A. (2012). Specificity of Dnmt1 for Methylation of Hemimethylated CpG Sites Resides in Its Catalytic Domain. Chem. Biol..

[77] Symmank J., Zimmer G. (2017). Regulation of Neuronal Survival by DNA Methyltransferases. Neural Regen. Res..

[78] Aapola U., Liiv I., Peterson P. (2002). Imprinting Regulator DMNT3L Is a Transcriptional Repressor with Histone Deacetylase Activity. Nucleic Acids Res..

[79] Deplus R. (2002). Dnmt3L Is a Transcriptional Repressor That Recruits Histone Deacetylase. Nucleic Acids Res..

[80] Jackson D.C., Ultsch G.R. (2010). Physiology of Hibernation under the Ice by Turtles and Frogs. J. Exp. Zool. Part A Ecol. Genet. Physiol..

[81] Wijenayake S., Storey K.B. (2016). The Role of DNA Methylation during Anoxia Tolerance in a Freshwater Turtle (*Trachemys Scripta Elegans*). J. Comp. Physiol. B Biochem. Syst. Environ. Physiol..

[82] Biggar K.K., Storey K.B. (2012). Evidence for Cell Cycle Suppression and MicroRNA Regulation of Cyclin D1 during Anoxia Exposure in Turtles. Cell Cycle.

[83] Biggar K.K., Storey K.B. (2017). Exploration of Low Temperature MicroRNA Function in an Anoxia Tolerant Vertebrate Ectotherm, the Red Eared Slider Turtle (*Trachemys Scripta Elegans*). J. Therm. Biol..

[84] Watts A.J., Storey K.B. (2019). Hibernation Impacts Lysine Methylation Dynamics in the 13-Lined Ground Squirrel, *Ictidomys Tridecemlineatus*. J. Exp. Zool. Part A Ecol. Integr. Physiol..

[85] Hawkins L.J., Storey K.B. (2018). Histone Methylation in the Freeze-Tolerant Wood Frog (*Rana Sylvatica*). J. Comp. Physiol. B Biochem. Syst. Environ. Physiol..

[86] Wijenayake S., Hawkins L.J., Storey K.B. (2018). Dynamic Regulation of Six Histone H3 Lysine (K) Methyltransferases in Response to Prolonged Anoxia Exposure in a Freshwater Turtle. Gene.

